# Progressive mobility program and technology to increase the level of physical activity and its benefits in respiratory, muscular system, and functionality of ICU patients: study protocol for a randomized controlled trial

**DOI:** 10.1186/s13063-018-2641-4

**Published:** 2018-05-10

**Authors:** Debora Stripari Schujmann, Adriana Claudia Lunardi, Carolina Fu

**Affiliations:** Department of Physical Therapy, Communication Sciences & Disorders, and Occupational Therapy, School of Medicine, University of São Paulo, Rua Cipotânea, 51, Cidade Universitária, Sao Paulo, SP Brazil

**Keywords:** Early mobility, Rehabilitation, Critical care

## Abstract

**Background:**

Enhanced mobility in the Intensive Care Unit (ICU) could minimize the negative effects of critical illness, such as declines in cognitive, muscular, respiratory, and functional capacity. We aim to compare the functional status at ICU discharge of patients who underwent a progressive mobilization protocol versus patients who received conventional physiotherapy. We also examine the level of physical activity in the ICU, the degree of pulmonary and muscle function, and the length of stay to analyze correlations between these variables.

**Methods:**

This is a protocol for a randomized controlled trial with blind evaluation. Ninety-six ICU patients will be recruited from a single center and randomly assigned to a control group or an intervention group. To determine the level of protocol activity the patient will receive, the patients’ ability to participate actively and their muscle strength will be considered. The protocol consists of five phases, ranging from passive therapies to walking and climbing stairs. The primary outcome will be the functional status at ICU discharge, measured with the Barthel Index and the ICU Mobility Scale (IMS). Measured secondary outcomes will include the level of physical activity, maximal inspiratory and expiratory pressures, forced expiratory volume in 1 second, maximum voluntary ventilation, handgrip strength, surface electromyography of the lower limb muscles, and results of the Timed Up and Go and 2-Minute Walk tests. Evaluations will be made within 2 days of ICU discharge except for the level of activity, which will be evaluated daily. Physiological variables and activity level will be analyzed by chi-square and *t* tests, according to the intention-to-treat paradigm.

**Discussion:**

Mobility and exercise in the ICU should be undertaken with intensity, quantity, duration, and frequency adjusted according to the patients’ status. The results of this study may contribute to new knowledge of early mobility in the ICU, activity level, and varying benefits in critical patients, directing new approaches to physiotherapeutic interventions in these patients.

**Trial registration:**

Recruitment will begin in February 2017, and the expected completion date is August 2018. Patients are already being recruited.

ClinicalTrials.gov, ID: NCT02889146. Registered on 3 March 2016.

**Electronic supplementary material:**

The online version of this article (10.1186/s13063-018-2641-4) contains supplementary material, which is available to authorized users.

## Background

A large number of patients who survive hospitalization in the Intensive Care Unit (ICU) experience negative effects of critical illness, even after stabilization of the condition, presenting cognitive, psychological, and physical changes as the main morbidities. In a prospective cohort study of 1075 patients 5 months after discharge, 48% of patients needed help in at least one activity of daily life [1]. Data show that 1 year after their stay in ICU, patients still reported muscle weakness, loss of muscle mass, and fatigue, and half of those patients had not yet returned to occupational tasks [2]. Even after 5 years, some of these changes persisted in most patients [3]. In another study, quality of life was evaluated at 1 year and 4 years after hospital discharge, with results indicating a decrease in quality of life in surviving patients due mainly to a decrease in physical function [4].

These medium and long-term consequences originate at the beginning of hospitalization and are related to changes that occurred during the stay in the ICU. In this period, patients may experience a period of inactivity and prolonged rest resulting in immobility, related to changes in various systems of the body. In the respiratory system, the main consequences of this immobilization are atelectasis, mechanical ventilation (MV), and hospital-associated pneumonia as well as delayed removal of the MV due to muscle weakness, decreased vital capacity, and residual volume [5, 6].

Studies of immobility in bed have shown a decrease in strength and muscle mass with variations similar to the losses experienced during ICU stays, with a decline of 1–1.5% per day and up to 50% of total muscle mass in 2 weeks [5]. In healthy people, immobilization has been shown to induce muscle atrophy, characterized by decreased protein content, fiber diameter, fatigue resistance, and strength [7]. Signs of cardiovascular dysfunction are observable within 3 to 4 days of immobility in the bed [8]. These effects, seen in healthy, bed-restricted patients may be potentially relevant for ICU patients who also present with fluid loss, as evidenced by a decrease in plasma volume of 10–20%. This decrease contributes to postural hypotension and tachycardia as well as decreased systolic volume, ejection volume, cardiac output, and oxygen uptake [9, 10].

In the ICU, in addition to immobility, muscular dysfunction is common due to multiple factors such as inflammation, the use of pharmacological agents (corticosteroids, muscle relaxants, or antibiotics) and the presence of neuromuscular syndromes associated with critical illnesses: critical illness myopathy and polyneuropathy [6, 8, 11–13]. Peripheral muscle weakness has been reported in 25–33% of patients undergoing MV for 4 to 7 days and in 60% of patients with respiratory failure [14, 15].

In light of these facts, interventions that encourage greater mobility in and out of bed during hospitalization are necessary to avoid or minimize losses associated with immobility and inactivity. The European Respiratory Society and the European Society of Intensive Care recommend starting these interventions as early as possible in critical patients, which can be done through both passive and active exercises [16]. Recent studies have indicated that early activity for ICU patients is safe and feasible, and results in a larger number of patients who leave the ICU functionally independent. Of all patients discharged from the ICU, those who underwent early mobilization left with fewer complications related to immobility and with improved functionality [17–19].

Among the benefits described in the academic literature on early mobility are increased days free of MV, reduced incidence of ventilator-associated pneumonia, reduced skin lesions, reduced days of hospital and ICU stay, decreased duration of delirium, and improved physical function at hospital discharge [5, 17, 19, 20].

There is no clear consensus on the ideal prescription for patients in ICU; however, the American College of Sports Medicine proposes a basic program [21]. The program should include a prescription of intensity, volume, and frequency for each patient to optimize the benefits from physical activity, even for ICU patients and those with respiratory insufficiency [7]. However, there are no guides or well-established practices for early and progressive mobility in the ICU. Determining the optimal initiation and progression of physical exercises can enhance beneficial effects of the protocol. Studies of current mobility protocols are still scarce in the academic literature of physical activity.

Rehabilitation programs within the ICU have been proposed, but the academic literature describes some limitations to early mobility in critical patients such as a lack of knowledge among professionals about early mobility, sedation practices, and insufficient numbers of professionals or equipment [5, 22]. Since a shortfall of professionals and a lack of equipment are cited as barriers to rehabilitation programs, the use of technology has emerged as a possible way to provide such services. Thus, the introduction of rehabilitation technologies, such as equipment for strength exercises, neuromuscular electrical stimulation, cycle ergometers, and equipment for walking can be an important factor in improving muscle strength, cardiopulmonary function, and functionality for patients in intensive care. Virtual reality has been suggested as a tool to improve certain cognitive functions. This is because exercise linked to virtual reality is able to offer demands that include multitasking, inhibition, taskswitching, short-term/working memory, and intelligence. Games can offer cognitive work since they generate cognitive demands such as: plan movements in order to hit targets, response inhibition, divide attention between tasks, sustain attention, follow visual and auditory cues, identify stimulus and decide quickly [23]. In the ICU, the use of these technologies, along with mobility programs, requires a thorough evaluation of the potential benefits and the safety, practicality, and efficacy.

Therefore, we created an early and progressive mobilization program based in four areas—muscle, cardiovascular, respiratory, and cognitive activities—to promote appropriate functional capacity and performance. The program includes exercises to maintain or recover basic function and to integrate these systems, preserving or recovering patients’ functionality even during hospitalization in the ICU.

The study’s hypothesis is that a progressive mobilization protocol and the use of technology is superior to conventional physiotherapy in promoting activity levels in the ICU and provides greater functional capacity at ICU discharge. Given evidence that points to the benefits of early mobilization, we chose to use conventional physiotherapy with the control group.

## Methods

### Aim

The primary goal of the study will be to compare the functional discharge status of ICU patients who undergo conventional physiotherapy with ICU patients who receive the progressive mobilization protocol. A secondary goal will be to assess and compare levels of physical activity in the ICU, length of stay, and pulmonary and muscle changes in patients who receive conventional physiotherapy and those who undergo the progressive mobilization protocol.

### Design

This study uses a protocol for a randomized and controlled trial with blind evaluation.

### Setting

The study will be conducted in the general ICU of a tertiary level university hospital (Hospital das Clínicas at the University of São Paulo) in São Paulo, Brazil. The unit has a physiotherapist available at all times, and patients receive physical therapy twice a day. The patients receive respiratory care and mobilization from physical therapy professionals. There are no physiotherapy protocols in the unit; the protocol described in this article will be added to the physiotherapy routine for this research. The ICU is a general unit that receives patients with all kinds of illness as well as clinical and surgical patients. The average stay in the ICU is 12 days, and there are 14 beds.

### Participants

Participants will be patients admitted to the ICU who are 18 years old or older with a Barthel Index equal to 100 points before hospitalization, who have not been transferred from another hospital, and who meet the criteria for inclusion in the progressive mobilization program will be enrolled in the study. The criteria for for initiation and continuity the progressive mobilization program follow the safety consensus described in the academic literature [19] and some points are as follows: respiratory rate (RR) ≤ 35 breaths per min, absence of cardiac arrhythmias or acute ischemia, heart rate (HR) between 50 and 140 beats per min, without the use of vasoactive drugs or without dose increase, mean arterial pressure (MAP) between 60 and 120 mmHg, absence of active bleeding, and no prescription of bed rest. Patients with MV with inspired fraction of inspired oxygen (FiO_2_) ≤ 60% and positive end-expiratory pressure (PEEP) ≤ 10 cmH_2_O are also eligible to participate.

Patients to be excluded include patients with neurological diagnoses associated with motor alterations, who refuse to carry out the proposed exercises or ask to withdraw less than 3 days after beginning the protocol, with contraindications for movement, those with pre-existing amputations or who undergo amputation upon hospitalization, and those who die before the final evaluation will be excluded from the study.

After admission to the ICU, patients will be evaluated at all times of the day regarding the inclusion criteria in the program. Patients will be evaluated within 2 days of admission to the ICU to participate in the study. If they do not present the criteria for initiating a mobilization program in that period [19], they will not be randomized and will not be included in the study.

### Details of the intervention and control

The study will consist of two groups: control and intervention. Patients will be randomized into groups using a computer program after evaluation of the inclusion criteria. Both groups will receive respiratory care according to the routine of the ICU and will receive physiotherapy twice daily. The ICU staffs a physiotherapist at all times. Patients in the intervention group will receive conventional physiotherapy in the morning and perform the research protocol in the afternoon period, thus receiving physiotherapy twice a day. Patients in the control group will also receive physiotherapy twice a day, performing conventional physiotherapy in both the morning and afternoon.

#### Control group

The control group will perform the conventional treatment offered by the unit physiotherapists. The unit physiotherapist will not have access to the protocol and equipment used, and the exercises that patients perform will be at the physiotherapist’s discretion. Conventional therapy consists of passive, active assisted, and active mobilization as well as bed positioning, bedside and armchair transfers, orthostatism, and ambulation; however, the type of therapy will not be defined in advance, but instead will be at the discretion of the attending physiotherapist and without a pre-established routine. No technological equipment will be used for this group, as these elements are not part of the normal service.

#### Intervention group

**Fig. 1:**
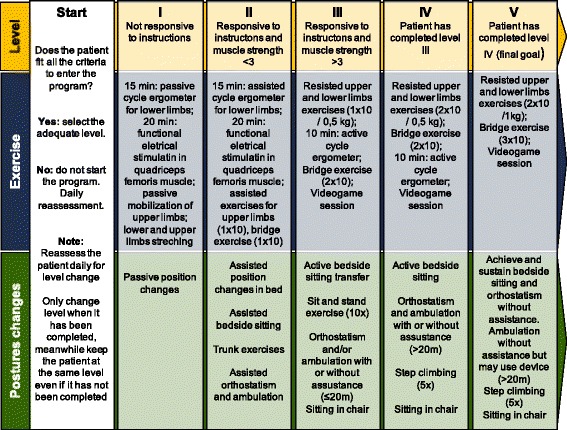
Progressive mobilization program

The intervention group will undergo a program of early and progressive mobilization (Fig. 1) created by the research team, based on data in the academic literature. As maintenance of functionality seems to depend primarily on the muscular and cardiorespiratory systems, the program is designed for rehabilitation focused on these systems. The protocol describes exercises, techniques, and apparatus for maintenance of muscle length, maintenance and gain of muscle strength, aerobic exercises, exercises aimed at reeducation of gait, and cognitive components.

The patients will undergo the prescribed program therapy at the appropriate level for each patient, chosen based on the physiotherapist’s evaluation and evolving within the protocol as described below. The program should be started within 48 h of ICU admission. The patient will be included in the program throughout their stay in the ICU and will be assisted by a specific physiotherapist to apply only the prescribed protocol.

According to the program description (Fig. 1), the patients in the intervention group will have equipment and technologies for the therapy. These devices are a functional electrical stimulator (Fesmed II, Carci®, São Paulo, Brazil), dumbbells, shin guards, a Motomed Letto® active and passive cycle ergometer (RECK Company, Bremen, Germany), a fixed walker, a LiteGait 300MX-30® ambulatory device (Mobility Research, Tempe, AZ, USA), and a Wii Fit® video game with sitting and standing games (Nintendo®, Redmond, WA, USA).

If, during hospitalization, the patient evolves with criteria that indicate that any level of activity has been performed for more than 2 days, they will be excluded from the study.

The five levels of the early and progressive mobilization program (Fig. 1) are described below.

#### Level I

Patient characteristics: not responsive to commands.

Physical exercises: passive cycle ergometer for lower limbs (15-min duration) with 35 revolutions per min, passive upper limb mobilization with ten repetitions of flexion and abduction of shoulders and triple hip flexion, and tibialis ischial stretching and simultaneous functional electrical stimulation (FES) in the right and left quadriceps muscles. FES will be set with the following parameters: frequency (R) 60 Hz, pulse width (T) 250 ms, on time of 4 s and off time of 8 s, applied for 20 min.

Postural changes: passive transfer of decubitus.

#### Level II

Patient characteristics: responsive to commands, muscle strength (MS) less than three for hip flexors and extensors according to the Medical Research Council (MRC) Scale [32].

Physical exercises: assisted exercises of upper limbs (one series of ten repetitions), cycle ergometer preferentially assisted in the lower limbs with a duration of 15 min, FES in the quadriceps muscles (15-min duration), bridge exercise (one series of ten repetitions), video game session of 15-min duration.

Postural changes: active bed transfer, assisted bedside sitting, trunk control exercises, attempted orthostatism, and assisted walking with specific technologies.

#### Level III

Patient characteristics: responsive to commands, MS greater than or equal to three for hip flexors and knee extensors according to the MRC Scale.

Physical exercises: resistive exercise of shoulder abductor, elbow flexor, hip flexor, and knee extensor muscles (one series of ten repetitions with a load of 0.5 kg), active cycle ergometer in lower limbs (15-min duration), bridge exercise (one set of ten repetitions), video game session lasting 5 min.

Postural changes: active transfer to a seated position at bedside, sit and lift training (ten repetitions), orthostatism and walking with or without assistance for a distance less than 20 m, sitting in an armchair.

#### Level IV

Patient characteristics: responsive to commands, MS greater than three according to the MRC Scale, completion of level III of the program.

Physical exercises: continuation of resisted exercises (two sets of ten repetitions with a 0.5-kg load), bridge exercise (one set of ten repetitions), video game session for 5 min.

Postural changes: actively sitting at the bedside, orthostatism and walking with or without assistance for a distance greater than 20 m, training in going up and down steps, sitting in an armchair.

#### Level V

Patient characteristics: responsive to commands, MS greater than three according to the Medical Research Council (MRC) Scale, completion of level IV of the program.

Physical exercises: continuing resisted exercises of upper and lower limbs (one series of ten repetitions with a load of 1.0 kg), bridge exercise (one series of ten repetitions), video game session lasting 5 min.

Postural changes: independent maintenance of bedside sitting, active orthostatism, and independent walking with or without aid for a distance greater than 20 m, climbing stairs, and sitting in an armchair.

#### Choice of activity level and criteria for progression in the mobilization program

The program consists of different levels of mobilization with progressive levels of activity, intensity, and types of exercise. To determine the appropriate level, the methods used to determine the appropriate level for each patient will be the patient’s ability to understand and their MS. The patient’s capacity to understand will be evaluated through requests to follow simple commands, such as “Put your hand on your head, bend your legs, open and close your eyes.” MS at the beginning of the program will be assessed using the MRC Strength Scale. A score of 3 points will be used as a cut-off to determine whether the patient can perform the movement alone and against the action of gravity. Patients capable of these movements will progress to the next level.

Thus, if a patient meets the criteria to enter the program, the next step will be to determine the activity level at which he or she will start the protocol. The first criterion is whether the patient can respond to commands; patients who are unresponsive will enter the program at level I. Patients who can follow commands will begin at levels II, III, IV, or V. To determine the specific level of each patient, MS is measured, with a cut-off score of 3 on the force scale. Patients with MS less than 3 will begins the program at level II, patients with MS of 3 or greater will begin at level III. The difference between level I and the other levels is the ability to understand commands, and the differences between the other levels depend on MS. After completing a level, the patient can move to the next level.

Patients will be reassessed daily to determine whether they can advance to the next level. To progress from level I to level II, the patient should have improved consciousness and be able to respond to commands. If MS is less than 3, the patient will enter level II. If the patient’s MS is equal to or greater than 3, they can advance directly to level III.

From level II onwards, patients can advance by one level when they can perform all the exercises prescribed for that level. Therefore, at levels III, IV, and V in the early and progressive mobilization program, the patient must be able to perform all of the physical exercises and postural changes at their present level or they will remain at the same level for the following day. When patients can complete all of the activities, they advance to the next level on the following day.

Patients in both the control and intervention groups will be monitored throughout the therapy for hemodynamic and respiratory stability. Therapy will be discontinued if the RR is above 35 breaths/min, the MAP is below 60 mmHg or above 120 mmHg, the HR is below 60 bpm or above 140 bpm, or the patient experiences discomfort or unrelenting pain during the therapy.

#### Patient assessment procedures

At the time of admission to the ICU and up to 2 days after admission, the patient will be examined against the inclusion criteria. The patient’s functionality in the 2 weeks before admission will be evaluated with the Barthel Index, which will be assessed by directly questioning the patient or their caregiver if they are unable to respond. Evaluation will include hemodynamic, respiratory, and cardiac stability for the safe practice of early mobility, and the absence of exclusion criteria will also be assessed. The patient will then be randomly placed in the control group or the intervention group.

After the patient is enrolled in the study, the initial variables will be collected, consisting of data related to the patient and their hospitalized and pre-hospitalization levels of functionality. At that time, an accelerometer will be placed on the patient to monitor their level of physical activity during the entire stay in the ICU.

Data on the patient’s hospitalization and the evolution of their condition, as well as the level of physical activity performed, will be collected during the entire stay in the hospital.

**Fig. 2:**
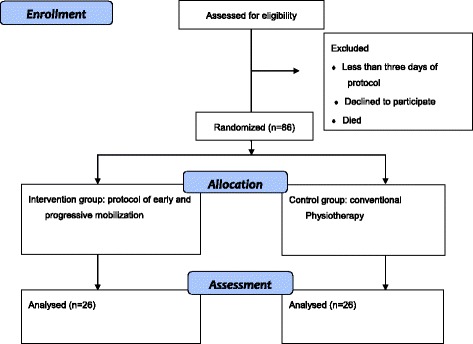
Flowchart of the study

Upon discharge from the ICU or within 2 days of discharge, all patients will undergo a complete evaluation of their respiratory, muscular, and functional systems through methods already described in the academic literature and performed by a blind evaluator. The accelerometer will be removed, and the data will be analyzed in a dedicated computer program. Figure 2 shows a flowchart presenting the methods used in the study.

Patients in both groups will use an Actigraph wGT3X-BT accelerometer (ActiGraph LLC®, Pensacola, FL, USA) throughout the length of their hospital stay, a tool which analyzes their position and level of activity. The accelerometer will be placed on the dominant lower limb using a bandage and will not be removed until discharge, allowing information on the patient’s physical activity to be collected objectively. The evaluation of the intensity of the exercises performed during ICU stay in both groups will be performed through the parameters evaluated by the accelerometer. This instrument is able to identify the intensity of the exercise performed, and for analysis we will divide it into mild, moderate and intense or absence of activity. Exercise intensity will also be monitored and evaluated by the Borg Scale at the end of each physical therapy session. At the end of the ICU stay, the equipment will provide data such as the exercise intensity and time spent lying down, sitting, and standing [24].

The following data concerning hospitalization will be collected: use of vasoactive drugs, use of corticoids or neuromuscular blockers, hypoglycemia, the presence of sepsis, surgery, hemodialysis, use of invasive and non-invasive MV, tracheostomy, and the number of days spent in the different therapies that were evaluated.

Evaluation of the respiratory system, muscular system, and functionality after ICU discharge will be performed by a blind evaluator using methods previously described in the academic literature. These data will be correlated with the physical activity data obtained from the accelerometer.

The respiratory evaluation will be performed using the maximal inspiratory pressure test with the use of a manovacuometer (Wika Corporate®, Klingenberg, Germany), while evaluation of pulmonary function will be done through spirometry, assessing the forced expiratory volume in 1 second (FEV_1_) and maximum voluntary ventilation [25] with a Datospir – Micro C digital spirometer (Sibelmed®, Barcelona, Spain).

The muscular evaluation will be performed by measuring MS with a handgrip dynamometer (Jamar Enterprises LDT®, Hoddesdon, United Kingdom) [26]. Muscle activity will be measured by electromyography of three muscles in the lower limbs—the anterior tibial, medial gastrocnemius, and vastus lateralis muscles—using a Miotool Miotec device (Miotec Equipamentos Biomédicos Ltda®, Porto Alegre, Brazil). The patient will perform the voluntary contraction three times in each muscle, accompanied by an electrogoniometer (Miotec Equipamentos Biomédicos Ltda®, Porto Alegre, Brazil) [27, 28].

Muscular function and mobility will be evaluated using the Timed Up and Go test [29] and the 2-Minute Walk test [30].

Functionality will be evaluated using the Barthel Index [31], measured by directly questioning the patient and by using the ICU Mobility Scale (IMS) [32].

The demographic data and initial evaluation of the patient will be collected on a data sheet. The patient’s physical activity data will be collected by the accelerometer and stored in the equipment’s software, and will later be included in the general tabulation of patient data. The physical evaluation data (dynamometry, electromyography, respiratory MS, and spirometry) will be collected on the same data sheet.

#### Follow-up for outcome assessment

A longer-term follow-up will be performed for functional status by the Barthel Index. This scale will be reapplied at the time of hospital discharge, after 3 months and 1 year of hospital discharge. At these times the Barthel Index will be re-evaluated by phone contact.

### Primary and secondary outcome measures and assessment points

The outcome variables of the study will be respiratory, muscular, and functional variables; the length of stay in the ICU; and analyzed variables of physical activity, measured by the accelerometer.

The primary outcome will be the functional status at discharge from the ICU. The functional status is the difference between baseline and final score on both the Barthel Index and the IMS. Therefore, at discharge from the ICU, the Barthel Index and IMS will be redone, and the quantitative final score on those scales will be considered as the primary endpoints of the study. The secondary outcomes will be the respiratory and muscular variables and the number of days of hospitalization in the ICU. Further secondary outcomes will be the variables of physical activity performed during this period, which include an analysis of the intensity levels of the exercise to which the patient was submitted and the percentage of time spent sitting, lying, and standing.

**Fig. 3:**
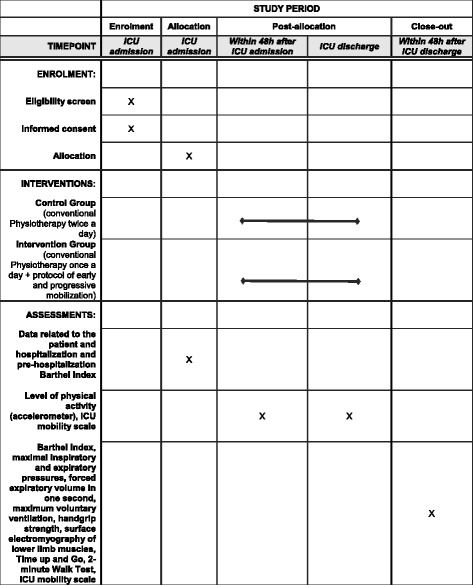
Standard Protocol Items: Recommendations for Interventional Trials (SPIRIT) Figure, describing the schedule of the study

For both groups, the occurrence of any adverse events related to motor therapy, such as respiratory or hemodynamic instability, accidental removal of invasive devices, falls, or pain will be recorded. Figure 3 illustrates the schedule of enrollment, allocation, intervention, and assessment of the study.

### Recruitment procedures

Patients will be recruited directly in the ICU at the time of admission to the unit. The criteria for inclusion in the study and the signing of informed consent for inclusion in the study will be analyzed. Data will be assessed only by the researchers and kept confidentially. Patients have been recruited since February 2017, and the expected completion date of the study is August 2018.

### Randomization procedures

Patients will be allocated to each group through computerized randomization. It will be a study of superiority, conducted in a single center. The patient allocation will be concealed, and the randomization of the individual will be performed by a researcher who is not involved in enrolling the participants, assigning them to groups, or performing follow-up measurements. This researcher will keep the allocation hidden in sealed brown envelopes within a box, which will be opened individually only at admission to the ICU when the participant is enrolled in the study. Individuals will be distributed between two groups. Only the researcher responsible for conducting the protocol will know how the participants are distributed.

### Masking/blinding

This is a blind evaluator study. The evaluator will not know the group to which the evaluated subject was assigned. Therefore, the patients will be instructed not to report their activity during hospitalization, and the equipment used in the protocol will be removed from the patient’s bed so that the evaluator cannot see the equipment that was used. The evaluator will also have no access to the tabulation of the split group data and will record the evaluation data on a separate worksheet.

### Statistical analysis including sample size calculations

Analyses for all aims will be performed according to the intention-to-treat paradigm. Comparative analysis between the control group and the intervention group will be performed for all outcome variables. For the comparison of the physiological variables and the levels of activity between the groups, a statistical test will be used to compare the two independent groups. For parametric data, the *t* test will be used.

To calculate the sample size for the primary outcome, we based the design on results found in Schweickert (2009) for a clinical trial. To an estimated expected difference in means in the primary outcome (Barthel Index score) of 13 points and a standard deviation of 22 points. Considering a statistical power of 80% and an alpha error of 0.5, we found that the number of subjects should be 48 per group, totaling 96 patients in the study.

## Discussion

The main objective of this protocol will be to compare the use of an early and progressive mobility program designed to increase the level of physical activity with a conventional therapy program and assess its benefits for the respiratory, muscular, and functional systems of patients in the ICU. Objective descriptions of the intensity of exercise performed in the ICU are scarce in the academic literature.

Physiotherapy in clinical practice differs greatly in character between care services. Variation in the supply and types of treatments available within the ICU may be the main difficulty in achieving greater benefits, and standardization with early and progressive programs, with the patient being included at most appropriate level of the program (i.e., the maximum the patient can do at that moment), may be the key to better outcomes. Other procedures used in the ICU, such as weaning from MV, weaning from sedation and therapies for sepsis have previously been addressed and are implemented using defined protocols [18].

A study conducted in ICUs in Australia showed that, although the vast majority of physiotherapists routinely prescribed exercises in the ICU, practices varied widely across the country, and it was also found that the results of the therapies were not measured. These results led to the conclusion that future research is necessary for adequate and improved exercise prescription in ICUs [33].

As with other therapies, mobility and exercise in the ICU should be planned in terms of intensity, quantity, duration, and frequency. It is important that physiotherapy in the ICU also has directions and protocols for these characteristics [5]. Studies of current mobility protocols are still scarce, especially regarding physical activity characteristics, such as exercise intensity [13].

We hope that the early and progressive rehabilitation program will result in a higher level of physical activity during ICU admission and thus will also result in improved functional indexes and muscle and respiratory functions at ICU discharge. Also, we hope that our results will contribute to scientific knowledge about the benefits of physical activity in the ICU and help to direct physiotherapeutic activity in this environment, bringing benefits to patients and the health care system (Additional file 1).

### Trial status

Trial registration: ClinicalTrials.gov

Registration number: NCT02889146. Registered on 3 March 2016.

The trial commencement was February 2017. It is currently recruiting patients and anticipated completion date is July 2018.

## Additional file

Additional file 1: SPIRIT 2013 Checklist: Recommended items to address in a clinical trial protocol and related documents. (DOC 122 kb)

## Abbreviations

FES: Functional electrical stimulation
FEV_1_: Forced expiratory volume in 1 second
FiO_2_: Fraction of inspired oxygen
HR: Heart rate
ICU: Intensive Care Unit (ICU)
IMS: ICU Mobility Scale
MAP: Mean arterial pressure
MRC: Medical Research Council
MS: Muscle strength
MV: Mechanical ventilation
PEEP: Positive end-expiratory pressure
RR: Respiratory rate
